# Vaso‐occlusive crisis in a patient with sickle cell trait and COVID‐19

**DOI:** 10.1111/jcmm.16948

**Published:** 2021-10-23

**Authors:** Luiz A. R. Freitas, Lilian V. S. Carvalho, Jonathan L. M. Fontes, Cassiana S. Souza, Reginaldo B. Santos, Lara T. Cardoso, Claudio P. Figueira, Milton S. Fonseca Neto, Rafael C. M. F. Dias, Manuela S. Solcà, Marilda S. Gonçalves, Setondji C. M. A. Yahouedehou, Ceuci L. X. Nunes, Geraldo G. S. Oliveira, Washington L. C. dos‐Santos

**Affiliations:** ^1^ Gonçalo Moniz Institute Oswaldo Cruz Foundation Salvador BA Brazil; ^2^ Federal University of Bahia Salvador BA Brazil; ^3^ Couto Maia Institute Bahia State Health Secretary Salvador BA Brazil

**Keywords:** COVID‐19, sickle cell crisis, sickle cell trait, splenic infarction, thrombosis

## CONFLICT OF INTEREST

The authors declare no conflict of interest.

## AUTHOR CONTRIBUTIONS

**Luiz Antonio Rodrigues Freitas:** Conceptualization (equal); Formal analysis (equal); Writing‐original draft (equal); Writing‐review & editing (equal). **Lilian Verena da Silva Carvalho:** Formal analysis (equal); Methodology (equal). **Jonathan Luis Magalhães Fontes:** Methodology (equal). **Cassiana Silva Souza:** Resources (equal). **Reginaldo Brito Santos Jr**.**:** Methodology (equal). **Lara Torres Cardoso:** Formal analysis (equal). **Claudio P Figueira:** Formal analysis (equal); Methodology (equal). **Milton Salomar Fonseca Neto:** Methodology (equal). **Rafael Carvalho de Moura Freire Dias:** Methodology (equal). **Manuela Silva Solcà:** Methodology (equal). **Marilda Souza Gonçalves:** Formal analysis (equal); Methodology (equal). **Sètondji Cocou Modeste Alexandre Yahouédéhou:** Formal analysis (equal); Methodology (equal). **Ceuci Lima Xavier Nunes:** Resources (equal); Writing‐review & editing (equal). **Geraldo Gileno Sá Oliveira:** Conceptualization (equal); Formal analysis (equal); Methodology (equal); Writing‐original draft (equal); Writing‐review & editing (equal). **Washington Luis Conrado dos Santos:** Conceptualization (equal); Formal analysis (equal); Funding acquisition (equal); Methodology (equal); Writing‐original draft (equal); Writing‐review & editing (equal).

Sickle cell trait (SCT) is a benign condition that only rarely, under hypoxic conditions, may result in microvascular complications that are normally seen only in homozygous patients. In COVID‐19, diffuse alveolar damage and endothelial activation contribute to tissue hypoxia. Studies comparing COVID‐19 outcomes in patients with HbAS and normal haemoglobin have shown no differences between groups or greater mortality rate in the former group.^1^, ^2^ Here, we present a case of a 63‐year‐old man, mixed‐race, married, resident of Salvador, Bahia‐Brazil. He presented at a reference infectious disease hospital with flu‐like symptoms, fever, odynophagia and fatigue that started seven days prior to admission and worsened over the previous 24 h, in association with lipothymia and dyspnoea. The patient had a history of arterial hypertension and used losartan. RT‐PCR was positive for SARS‐CoV‐2. Chest radiographs, on the 2nd and 4th days following hospitalization, revealed ground‐glass opacities predominantly in the lower lung segments that evolved to consolidations. As the patient required mechanical ventilation, he was transferred to the intensive care unit and orotracheally intubated due to severe hypoxemia, gas exchange PaO_2_/FiO_2_ < 100 mm Hg, and acid‐base and electrolyte disturbances. Sedation was optimized, and the patient was treated with dexamethasone, omeprazole 40 mg/day (intravenously, 24 h), and Ceftriaxone and Azithromycin. In addition, the patient was treated subcutaneously with unfractionated heparin 5,000 UI 12/12 h for the period of hospitalization and, mistakenly, with enoxaparin 40mg/day from the first to fourth hospitalization days. Noradrenaline and dobutamine were added to the treatment regimen as the patient showed hypotension and signs of poor perfusion. Gas exchange improved after 20 h of pronation; however, improvement was not maintained when the patient was returned to the supine position. The patient presented kidney failure requiring haemodialysis. Relevant clinical pathology data are shown in the Table 1. Bacterial blood cultures and screening for HIV, syphilis and viral hepatitis were all negative. The patient was afebrile, yet exhibited a tendency to hypothermia. He received a transfusion of concentrated red blood cells, and antibiotic therapy was supplemented with Meropenem. After presenting dark‐coloured vomit and nasal secretions, enoxaparin was suspended. On the 7th following hospitalization, in critical condition, he presented bradycardia, refractory hypotension and oxygen desaturation, evolving to asystole and death.

**TABLE 1:** Clinical pathology data

Test	Day of hospitalization						
	1st	2nd	3rd	4th	5th	6th	7th
Haemoglobin (g/dl)	11.7	11.2	10.2	10.4	10	9.1	8.2
WBC (×10^9^/L)	17,240^a^	23,680^a^	47,030^a^	57,190^a^	66,470^a^	64,830^a^	49,919^a^
Serum albumin (mg/dl)		3.0	2.8	2.2	2.2	2.1	2.0
AST (U/L)	237	3,252	6,244	2,425	1,111	653	852
ALT (U/L)	95	1,562	2,615	1,491	1,046	680	523
GGT (U/L)	67	67	100	108	105	114	147
AP (U/L)	296	341	657	753	639	711	798
CPK (U/L)		425	5,095	28.023	32,347		
CPKMB (U/L)		135	211	635	497		
TB (mg/dl)	0.97	2,30	2.06	4.57	5.58	5.82	6.47
DB (mg/dl)	0.10	1.39	1.40	3.22	3.94	4.04	4.52

Abbreviations: ALT, alanine aminotransferase; AP, alkaline phosphatases; AST, aspartate aminotransferase; CPK, creatine phosphokinase; CPKMB, CPK myocardial band; DB, direct bilirubin; GGT, gamma glutamyl transferase; TB, total bilirubin; WBC, White blood cells.

^a^ leukocytosis with left shift.

A minimally invasive ultrasound‐guided autopsy was performed, and tissue samples were collected from the heart, lungs, liver, spleen, kidney, skeletal muscle of the brachial biceps and skin. Tissue fragments were fixed in paraformaldehyde and processed for histological analysis.

**FIGURE 1:**
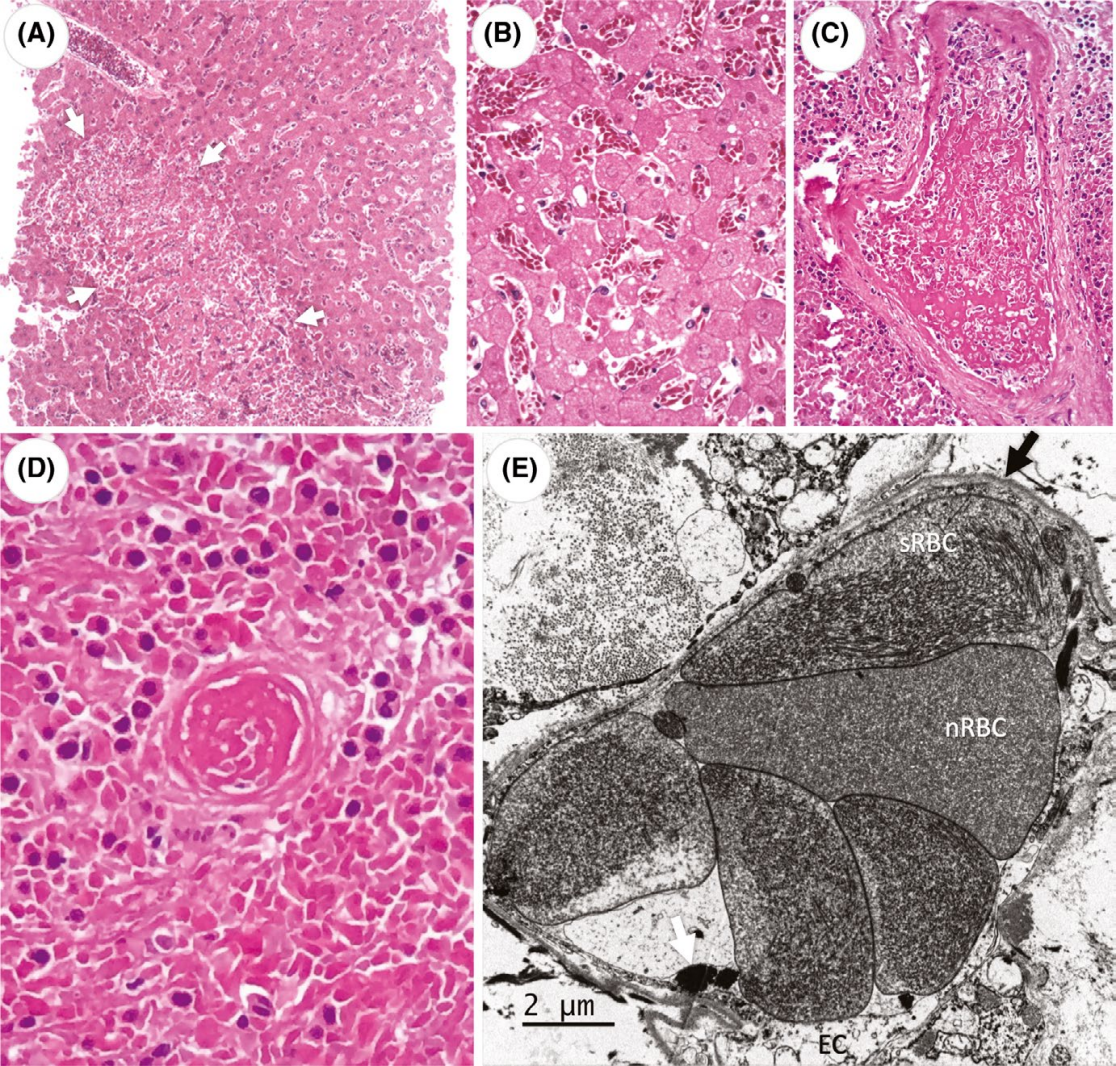
Vaso‐occlusive crisis in a patient with sickle cell trait and COVID‐19: A ‐ liver with large areas of coagulative necrosis (white arrows) with hepatocyte eosinophilia and nuclear loss (karyolysis, B). C ‐ Splenic arterial thrombosis with fibrin and incipient peripheral organization. D – Spleen coagulative necrosis with arteriolar thrombosis. E – Lung capillary packed with sickled red blood cell (sRBC) containing parallel filaments in the cytoplasm and rare normal red blood cells (nRBC) with smooth cytoplasm. Focal fibrin aggregates (white arrow) are present in the capillary lumen

The lungs showed lesions characteristic of an acute exudative phase of diffuse alveolar damage in all examined sections. Hypertrophic and hyperplastic type II pneumocytes were shed to the alveolar lumen and, in many sections, were mixed with hyaline membranes. Sickled red blood cells were observed in the lumen of congested alveolar capillaries (Figure 1E), as well as in foci of intra‐alveolar haemorrhage. The liver presented an overall preserved hepatic architecture. Areas of hepatic infarction characterized by extensive zone 3 coagulative bridging necrosis were observed (Figure 1A and B). In these necrotic areas, as well as in more preserved liver tissue, congested sinusoids containing clumps of sickled red blood cells were noted (Figure 1A and B). Canalicular biliary cholestasis was observed. No inflammation was detected in the portal tracts, nor in the parenchyma. All spleen histological sections showed extensive coagulative necrosis associated with blood vessel occlusion due to fibrinous thrombi in the arterial vessels and venules (Figure 1C and D). The splenic red pulp was suffused with sickled red blood cells. The white pulp was effaced.

Prompted by the findings of extensive thrombosis and infarction, as well as the occurrence of sickled red blood cells in some vessels, genomic analysis of the beta globin gene was solicited to investigate the presence of the beta S allele. Genomic DNA was obtained from paraffin‐embedded liver tissue using a QIAamp DSP DNA FFPE tissue kit (Qiagen, Hilden, Germany). PCR was carried out to amplify a segment of the beta globin gene using extracted DNA and specific primers. Amplified PCR product analysed by ethidium bromide agarose gel electrophoresis revealed a DNA band with the expected molecular weight. Following enzymatic clean‐up using the ExoSap‐It (ThermoFisher), the PCR product was sequenced in both directions using specific primers on an ABI3100 automated sequencer (Applied Biosystems).

The most relevant findings in the present case, in addition to diffuse alveolar damage characteristic of severe COVID‐19, were liver and splenic infarcts. The occurrence of splenic infarcts has been associated with a state of hypercoagulability in patients with COVID.^3^ Liver infarctions associated with COVID‐19 seem to be uncommon, as a search of the literature returned only a few cases of extensive liver infarcts.^4^, ^5^ The liver is an organ with dual circulation, in which venous blood from the portal vein primarily contributes to liver perfusion; additionally, venous blood mixes with arterial blood from the hepatic artery. Coagulative necrosis (ischaemic) in the liver occurs in zones 3 of the hepatic acini (perivenular) in cases of severe hypoxia. This is associated with severe congestive heart failure, sinusoidal occlusion syndrome, or sickle cell disease due to sinusoidal occlusion and hypoxemia. In the present case, while the patient did not have a reported history of sickle cell disease, the occurrence of liver and splenic infarcts associated with intense vascular congestion and clumps of sickled red blood cells in the absence of a previous history of haematological or vascular occlusive events raised the suspicion that the patient might have had undetected sickle cell disease or SCT. DNA extracted from liver fragments and molecular analysis at the sixth position of the beta globin chain revealed both GAG and GTG codons, thus confirming the suspicion of SCT. In contrast to the patients studied by Resurrection et al. (2021) and Singh et al. (2021), prior knowledge of SCT had been obtained, which may have had a favourable impact on the course of disease. Thus, the present findings, together with observations in the literature, suggest that the potential contribution of the genetic background of specific communities should be considered when treating patients with COVID‐19.

## Data Availability

The data that support the findings of this study are available on request from the corresponding author. The data are not publicly available due to privacy or ethical restrictions.
